# Stakeholders’ perspectives on lessons learnt from HPV mass vaccination in Nigeria

**DOI:** 10.1186/s12889-025-24418-0

**Published:** 2025-09-24

**Authors:** Daradara Kubura, Sidney Sampson, Grace Erekosima, Mahfus Dauda, Sunday Atobatele, Morenike Oni, Olugbemisola Samuel, Stallone Ngobua, Catherine Akpen, Grace Popoola, Chukwudinma Okoh, Al-Mustapha Mukhtar, Kelechi Nnaji, Emmanuella Nzeribe, Helen Ukoh, Imole Agunbiade, Fatima Mohammed, Hilary Okagbue

**Affiliations:** 1 Sydani Initiative for International Development, Sydani Group, Abuja, Nigeria; 2 Sydani Consulting, Sydani Group, Abuja, Nigeria; 3 Sydani Institute for Research and Innovation, Sydani Group, Abuja, Nigeria; 4 Department of Mathematics, Covenant University, Ota, Nigeria

**Keywords:** Campaign, HPV vaccination, Microplan, Myths, Technical working group, Strategy

## Abstract

**Background:**

To effectively reduce the risk of cervical cancer, Nigeria launched the HPV vaccine introduction using a phased approach; first phase in October 2023 across 16 states and the second phase across 21 states in May 2024. This initiative aimed to protect girls aged 9–14 from HPV infections, a primary cause of cervical cancer, through strategic planning and targeted implementation. This paper aims to share the Stakeholders' perspectives from nine states in Nigeria on lessons learnt during HPV vaccination that could be adopted in future campaigns and vaccinations.

**Methods:**

The study adopted a qualitative research design to evaluate the strategies deployed, challenges encountered, and lessons learned from the HPV Phase II vaccination campaign. Key informant interviews and in-depth interviews were conducted with officers of the healthcare agencies at the national, state, local government and ward levels and representatives of CSOs, who had key information about the strategies and those who implemented policies during the HPV mass vaccination in nine states in Nigeria. The qualitative data was validated, transcribed, coded and analyzed thematically.

**Results:**

Key components of the campaign included the establishment of fixed and temporary vaccination posts, strategic school and community engagement, and targeted communication efforts via social media, influencers, and local leaders. Cultural sensitivity, trained health workers, and data-driven micro plans were emphasized to enhance the program's effectiveness. The campaign successfully raised awareness and increased vaccination coverage across the nine targeted states. Effective strategies included leveraging cultural sensitivity, utilizing trained health workers, and employing data-driven micro plans to address logistical challenges. Despite these successes, significant challenges were encountered. Vaccine hesitancy fueled by misinformation about sterility, limited resources, security concerns, and difficulties accessing remote areas posed barriers to achieving wider coverage. Key lessons from the campaign highlighted the importance of early planning, effective rumor management through existing networks, and the crucial role of influential community members in amplifying the message and encouraging participation.

**Conclusion:**

The findings highlight critical lessons for future vaccination programs. Early planning, proactive social mobilization, effective rumor management, and leveraging community networks were instrumental in enhancing vaccine acceptance and coverage. These strategies serve as a model for overcoming barriers to public health interventions and should be prioritized in future efforts to improve HPV and other vaccination programs in Nigeria.

**Supplementary Information:**

The online version contains supplementary material available at 10.1186/s12889-025-24418-0.

## Introduction

Human papillomavirus (HPV) infection is the most prevalent viral sexually transmitted infection globally, causing cancer [1, 2]. Cervical cancer is the most common HPV-related disease, with more than 99% of cases caused by persistent genital high-risk HPV infection [3, 4]. According to the Cervical Cancer Action for Elimination (CCAE), one woman dies from cervical cancer every two minutes worldwide [5, 6]. Studies have shown that engaging in risky sexual behaviors, such as having multiple sexual partners, having a partner who has multiple sexual partners, and initiating sexual activity at a young age, increases the risk of HPV infection and the development of cervical cancer. [7–9]. Cervical cancer is a global public health problem, ranked as the fourth most common cancer in women, accounting for more than 660,000 new cases and 350,000 global deaths in 2022 alone [10]. Despite its global prevalence, the burden of cervical cancer is disproportionately distributed, with 90% of cervical cancer-related deaths occurring in low- and middle-income countries (LMICs), and 60% of those in Sub-Saharan Africa (SSA) [11].

Low- and middle-income countries have the highest rates of cervical cancer incidence and mortality [10]. The vulnerability of the disease in SSA region is particularly alarming, being the most common in twenty-three countries of the region [12, 13], recording 35 new cases and 23 deaths per 100,000 women annually [14]. This exposure can be attributed to multiple factors, including lack of access to cancer treatment, absence of effective population-level screening programs, poor awareness about prevention, inadequate cervical cancer information, inequitable access to health services, poverty and low socioeconomic status, stigma, late diagnosis, poor socio-cultural practices [11, 13, 14].

Nigeria bears a significant burden, having the highest incidence of cervical cancer cases and deaths in Africa, with 12,075 new cases and 7,968 deaths in 2020. The country ranks among the eight countries globally with the highest number of new cases [15, 16]. It is the second most common cancer among Nigerian women (after breast cancer), with an incidence rate of 11.9 cases per 100,000 women, in 2020 [17, 18]. Moreover, Nigeria accounts for 43.4% of the total annual new cervical cancer cases across West African countries, representing almost half of the region's burden, based on 2020 estimations [18].

The challenges facing Nigeria in addressing cervical cancer are complex and multifaceted. The country lacks an effective cervical cancer prevention policy, as demonstrated by the failure of the 2008–2013 and 2018–2022 National Cancer Control Plans (NCCP) to meet key targets, including 90% vaccination coverage and screening of half the eligible population [16]. Awareness levels are critically low, with studies revealing disappointing knowledge rates: a cross-sectional study in the southern and northern geopolitical zones found only 32.7% awareness among women aged 15–49 in the informal sector, another study among female students in Kano showed knowledge levels as low as 35.5% [19, 20]. Healthcare infrastructure compounds these challenges. Most health facilities lack adequate infrastructure to manage the disease effectively, and available equipment is insufficient. There is also significant geographic disparity, with management centers concentrated in urban areas while highly populated rural communities remain underserved or completely neglected [4].

To effectively reduce the risk of developing cervical cancer among its population, Nigeria launched the HPV vaccine introduction program using a phased approach in October 2023 [21]. Through the Multi-Age Cohort (MAC) campaign, the first phase of the rollout was kickstarted in 16 states out of the 36 states and FCT of the federation, during which single dose of the HPV vaccine was administered to girls between the age of 9 and 14 [22]. The free vaccine was made available to the eligible population via a five-day mass vaccination campaign that took place in communities, markets, schools and hard-to-reach settlements across the sixteen states, to ensure no eligible girl is left behind. Teams of well-trained, experienced and dedicated healthcare workers (HCWs) were deployed to vaccination sites that had been set up in 4,163 wards across the 16 states [23]. The successful rollout was made possible because of collaboration between the federal government, through the National Primary Health Care Development Agency (NPHCDA) and multilateral agencies such as, UNICEF, WHO, Gavi – the Vaccine Alliance, IVAC, etc. Gavi was instrumental in the procurement and distribution of the free vaccines; WHO assisted in training more than 30,000 HCWs on service delivery and end-to-end vaccine management, while IVAC engaged relevant traditional and community leaders to help combat the spread of misinformation and also trained Civil Society Organizations (CSOs) to mobilize the eligible girls for vaccination including young girls with disabilities, using their extensive networks [22, 23]. Despite its success, the program encountered significant challenges, there were issues related to parental consent, along with a national diphtheria outbreak that diverted attention from key stakeholders and posed a risk of further burdening an already overstretched healthcare system [22]. The second phase took place in May 2024 in similar fashion across 21 states, with a combined total of over 12 million girls vaccinated against HPV and HPV-related cancers, in both phases [24].

To achieve the global goals of health for all and the sustainable development goal (SDG 3), it is important to analyze the lessons from the intervention to guide future decisions and help achieve the action plan for 2030. This study is one of a few studies to qualitatively examine the HPV vaccine introduction program in Nigeria, focusing on nine targeted states. This research aims to explore the strategies deployed during the HPV vaccination project, the challenges faced, the measures taken to overcome them, and the achievements and innovative practices deployed at the state level during the program. Additionally, the study seeks to provide policy recommendations that could enhance the effectiveness of future health interventions and vaccine introduction programs in Nigeria.

## Methodology

### Study design

This study adopted a qualitative research design to explore the implementation strategies, challenges, mitigants, innovative practices, and lessons learned from the HPV vaccine introduction (phase 2) across nine Nigerian states. The qualitative approach was chosen for its ability to elicit rich, descriptive data and facilitate an in-depth understanding of the experiences, practices, and insights of stakeholders involved during the vaccine introduction.

### Study setting

The study was conducted in nine Nigerian states where Sydani Group a management consulting firm providing strategic consulting services to a wide range sectors, including health, education, agriculture etc., was contracted to provide support during phase 2 of the HPV vaccine introduction. These states are representative of 5 out of 6 geopolitical zones in the country giving diversity in social and cultural context. These states include: Cross River, Edo, and Delta (South-south), Kogi, Kwara, and Plateau (North-central), Ekiti (South-west), Ebonyi (South-east), and Katsina state (North-west). Detailed demographic and health infrastructure information about each state was documented to provide context for the study.

### Sample size

A total of 140 participants were targeted, comprising 30 for Key Informant Interviews (KIIs) and 110 for In-Depth Interviews (IDIs). These participants were carefully chosen because of their rich insights during implementation to ensure comprehensive coverage of the study objectives.

### Study participants, selections and strategy

Participants were selected through purposive sampling to ensure representation across all administrative levels involved in the HPV vaccine phase 2 introduction. The selection criteria were designed to include stakeholders with direct knowledge and rich insights into the vaccine campaign. The sample comprised individuals from national, state, local government areas (LGA), and ward levels. At the national level, 3 officers who oversaw the vaccine introduction campaign. At the state level, the participants included 3 high-level officers from the State Primary Healthcare Board/Agency, 3 officers each at the LGA and ward levels, and 2 Civil Society Organization (CSO) representatives from each of the nine state.

### Inclusion and exclusion criteria for selecting study participants

Participants were included if they played active roles in the HPV vaccination campaign, represented various levels of implementation (national, state, LGA, ward), and provided informed consent. However, individuals who did not meet the above criteria or were unavailable during the study period were excluded.

### Data collection methods and procedures

A semi-structured key informant interview guide was developed for the national and state-level officers, and a semi-structured in-depth interview guide was developed for participants at the local government and ward level. The study assessed participants' experiences and knowledge of activities conducted during the HPV vaccine introduction which includes planning and coordination, stakeholder engagement, financial management, supply chain, service delivery and health workforce, data management, supervision, and also the lessons learned and innovative practices adopted during the campaign. The data collection process was meticulously planned and executed. Participants were identified and contacted through the state-level technical assistants. Verbal consent was obtained after participants were briefed on the study’s objectives, confidentiality measures, and their rights. Interview sessions were held in participants' offices or other agreed-upon locations, audio was recorded and later transcribed verbatim. Research assistants received oversight training from experienced qualitative researchers to ensure adherence to study protocols. The data collection spanned 9 days in November 2024. The KII and IDI guides are available as supplementary materials.

### Reflexivity

Reflexivity was integrated into every stage of the research process to thoroughly assess the impact of the researchers on data collection, analysis, and interpretation. The multidisciplinary team, which included public health professionals, qualitative researchers, statisticians, independent researchers, and experts in vaccination campaigns, participated in systematic reflexivity practices. Team members recorded their assumptions, preconceptions, and developing insights throughout the fieldwork and analysis phases by utilizing reflexive journals. Regular discussions among researchers ensured scrutiny of emerging interpretations and mitigated individual biases through peer debriefing. Interviewers used non-leading probes to avoid imposing views on participants, ensuring neutrality and unbiasedness in data collection. Analyst triangulation was utilized, involving multiple researchers who independently coded the transcripts, with any differences addressed through consensus to improve interpretive balance.

### Quality assessment

This research ensured trustworthiness using Lincoln and Guba's framework [25]. Credibility was established through data triangulation (interviews, documents) and member checking. Prolonged engagement facilitated iterative interviews, enhancing understanding. Transferability was addressed via thick descriptions of state contexts and implementation. Confirmability was maintained through reflexive documentation (journals, audit trails) and analytic memos detailing thematic connections. Verbatim quotes grounded findings in participant experiences, further strengthening the research's rigor.

### Data analysis

Audio responses from participants were transcribed verbatim. Codebook was developed by the research team and the transcripts were imported into Dedoose software for both coding and analysis. Thematic analysis was employed to identify patterns and insights from the data, an initial coding was conducted in Dedoose to identify meaningful units of information, which were then grouped into broader categories within the software. Relationships between categories were examined to form overarching themes. These themes and sub-themes were synthesized to generate key insights aligned with the study objectives. The analysis emphasized extracting relevant quotes and narratives to provide a deeper understanding of the study findings.

### Ethical approval and consent to participant

The study protocol was reviewed and approved by the National Health Research Ethics Committee (NHREC). Confidentiality and data protection measures were upheld. Data were anonymized and stored securely in password-protected systems accessible only to authorized personnel. Participants were informed about the study’s purpose, potential risks, and benefits, and willingly gave their consent.

## Result

### Demographic

The demographics of the 140 participants reflect a gender-balanced and experienced immunization workforce across Nigeria, representing a national sample comprising 54% female and 46% male (Table 1). Most respondents (49%) are aged 41–50, followed by 36% aged 51–60, indicating a predominantly mid-to-late career group. In terms of education, the workforce is highly qualified, with 55% holding bachelor's degrees and 31% possessing postgraduate degrees, while only 14% have diplomas. Regarding tenure, 45% have been in their current roles for 2–5 years, and 23% have served for over a decade, reflecting both stability and long-term commitment. Stakeholder roles are diverse, including Immunization Officers (23%), Ward Focal Persons (21%), Health Promotion Officers (11%), and representatives from civil society organizations and monitoring and evaluation units. An additional 9% fall into the "other" category, comprising executive secretaries, directors, and managers.

**Table 1:** Socio-demographic Characteristics of Participants

Characteristic	Cross River	Delta	Edo	Ebonyi	Ekiti	Katsina	Kwara	Kogi	Plateau	National	Total (%)
*Gender*											
Male	5	4	4	13	3	15	4	5	10	2	65 (46)
Female	10	12	11	2	14	1	8	10	6	1	75 (54)
*Age Group*											
20–30	1	0	0	1	0	0	0	0	1	0	3 (2)
31–40	3	1	2	1	4	1	2	4	1	0	19 (13)
41–50	5	4	10	8	8	8	7	7	8	3	68 (49)
51–60	6	11	3	5	5	7	3	4	6	0	50 (36)
*Education*											
Diploma	1	1	1	1	1	8	0	2	5	0	20 (14)
Bachelor’s degree	7	8	10	9	7	7	9	11	9	0	77(55)
Postgraduate	7	7	4	5	9	1	3	2	2	3	43 (31)
*Years in Current Role*											
< 1 year	4	1	0	0	3	0	0	0	1	0	9 (6)
2–5 years	6	8	8	4	9	4	6	5	11	2	63 (45)
6–10 years	2	5	3	2	4	4	4	9	3	0	36 (26)
> 10 years	3	2	4	9	1	8	2	1	1	1	32 (23)
*Stakeholder Group*											
Immunization Officers	3	2	3	3	5	4	4	2	6	0	32 (23)
Cold Chain Officers	1	1	1	0	1	2	3	2	1	0	12 (8)
Ward Focal Person	4	4	4	3	2	4	2	3	3	0	29 (21)
Civil Society Organizations	2	2	2	2	2	2	2	2	2	0	18 (13)
State mobilization officers	0	0	0	0	0	2	1	3	1	0	7 (5)
Monitoring and Evaluation Officers	3	1	2	4	2	0	0	2	0	0	14 (10)
Health Promotion Officers	0	4	2	2	5	2	0	0	1	0	16 (11)
Others	2	2	1	1	0	0	0	1	2	3	12 (9)

### Strategies deployed during the HPV phase II vaccination across the nine targeted states.

The HPV Phase II campaign across the nine Nigerian states utilized diverse strategies to enhance vaccination coverage. Fixed and temporary posts were employed, with temporary posts deployed to schools, markets, and remote areas to reach more people, especially out-of-school girls. Schools were a major focus, engaging teachers as crowd controllers and among the vaccination teams to foster trust among students and parents. Communities were targeted using traditional leaders, town criers, and community-based mobilizers who provided localized advocacy and resolved logistical issues. Extensive use of social media and influencers helped disseminate information and counter misinformation. Cultural sensitivity was ensured by translating campaign messages into native languages, and local leaders and health workers familiar with the communities were engaged. Capacity building through training and strategic use of micro plans optimized the deployment of vaccination teams, ensuring data-driven decisions and adaptability in addressing challenges. These multifaceted approaches led to widespread awareness, acceptance, and significant coverage across the targeted states.

#### Pre-implementation

##### Technical working group

The Technical Working Group (TWG) was a comprehensive committee established to oversee the HPV vaccine introduction campaign at national and state levels. Comprised of EPI officers, partners, and an expanded group of stakeholders including religious leaders, educators, community representatives, and media, the TWG provided technical guidance, monitored progress, and addressed implementation challenges. The group created specialized sub-committees for logistics, sensitization, and awareness, ensuring effective resource allocation, coordination and security. Key responsibilities included preparing for vaccine rollout, dispelling misinformation, sensitizing communities about vaccine benefits, and coordinating communication strategies to increase vaccine uptake. The expanded TWG (eTWG) played a crucial role in bringing diverse perspectives and stakeholders together to support the successful implementation of the HPV vaccination program.

*“The HPV Technical Working Group is a working group that was established and guided on the principle of working together, comprises of both government and partners, and with one aim, and the aim is to coordinate the HPV vaccine introduction in the country” (KII/National HPVVI Focal Person)*

*“So aside that we had some subcommittee under Technical Working Group. There was the training working group, social mobilization/ACSM working group, vaccine and logistic working group. We also had M&E working group so we have the data and other things. We had the waste management working group in which the Ministry of Environment was part. So then we are meeting every day to see the progress of each working subcommittee.” (KII/KW/SIO)*

*“Yeah, majorly the responsibility of the HPV TWG is actually planning and coordination, planning of implementation across all key thematic areas ranging from demand generation, service delivery, logistics, human resource for health for both national and sub-national across the thematic areas of the HPV implementation.” (KII/Director ACSM)*

*“Despite all the rumors that aired previously before the uptake of the vaccine, you can see that during the campaign we didn't have any case of rejection because all these technical working group members they really worked and cooperated with us, even we also met with the WDC chairman. So, in fact all of us really worked as a team dispelling rumour and creating more awareness among the general public.” (IDI/EK/Ido LGA/LHE)*

*“So we had the Odionweres, the Enogies using their palace. Then some gave us their hunters as vigilantes that will follow us to security compromised area to go and do hit-and-run vaccination. They will tell us when to come to vaccinate, when it's safe for us to come, and when it's not safe.” (KII/ED/Ovia NE/LIO)*

##### Microplan (MP) development

The HPV vaccine introduction program was meticulously planned and executed through comprehensive microplanning and community engagement strategies. The microplan served as a critical roadmap, developed through a multi-level approach involving national, state, and local stakeholders, with rigorous data collection using GIS mapping and community-level collaboration. Stakeholders from all levels validated the microplan to ensure accuracy and alignment with ground realities. Community sensitization efforts were extensive, utilizing diverse communication channels including market-day events, religious venues, schools, radio programs, and formal communications to maximize awareness. Complementing these efforts, a systematic training cascade was implemented, with national-level officers training state-level teams, that trained local government and ward-level healthcare workers, ensuring that all personnel were equipped with the necessary knowledge and skills to implement the HPV vaccine program.

*“If you have a very good plan, you will capture everywhere. Without microplan, one will not know which settlements to visit for vaccination. But if you plan properly, from the first day to the last day, you know where you are going, you know how many girls you are targeting in a school… It allowed us to see the number of schools we had, then the number of girls in each school. It guided us on the number of vaccines we would need, the number of devices we will need, because if we hadn’t done that, definitely we might have had a shortage or excess…So micro-planning is the basic for any implementation” (KII/ED/SIO)*

*“After we developed our micro plan at ward level, we submitted it to the LGA level, where the STF, LGA facilitator and LGA team went through it and validated it. People from the state also came to validate it. Sometimes, we would take them to the communities to validate. Sometimes they will ask us to take them to the community leader's house to know exactly how we did the micro plan or we just sat and wrote it by ourselves.” (IDI/KT/Kurfi/Birchi/WFP)*

*“And after the micro-plans were developed, the states were supported to verify what the LGAs had developed. And after the validation the national also went to validate the microplan. So the states verified the micro-plans developed from the wards and submitted them to the LGAs. The states also collated these micro-plans and sent them to national. And then we did another round of validation to ensure that the process was well followed, and the quality of the micro-plans are good.” (KII/National HPVVI Focal Person)*

##### Stakeholder engagement/ACSM strategies

Strategies deployed to engage stakeholders included advocacy visits to key stakeholders, including local government chairpersons, traditional rulers, community leaders, religious leaders, and school administrators to explain the importance of the HPV vaccine and address misconceptions. Ministries of education, and women affairs were engaged to secure cooperation and support, established task forces, security agencies, and other key stakeholders were engaged to ensure smooth campaign implementation. The media was utilized to disseminate vaccine-related information, including translating jingles into local languages to reach diverse communities. Online campaigns through social media platforms like Facebook was conducted to reach younger audiences. Town announcers were engaged and equipped with megaphones and public address systems to inform communities about vaccination dates and locations, roadshows were organized to create awareness and draw attention to the campaign. Finally, vaccine champions were engaged at the ward level to mobilize communities, ensure community-wide awareness, minimize vaccine hesitancy, and enhance campaign participation, particularly among target populations.

*“Yes, yes. I think one other strategy that I love was the translation of the jingles in native languages. So, if I don’t understand English, if I don’t understand Hausa, I’m Ejagha man, some other person can be Birom, some other person can be Kataf. Of course, Jos is a cosmopolitan city. So, these jingles were, you know, translated, transcribed into these languages. And of course, people appreciated it.” (KII/PL/DDCI)*

*“So some of these ACSM activities is creating awareness within the communities and secondly is to debunk rumors, if there is also community mobilization in time of distribution of IEC materials and SBCC materials and you know sometimes a lot of things do take place as far as ACSM is concerned, and that is why we have a lot of achievement as far as the HPV campaign is concerned, they were a lot of community engagement activities and there is a lot of house to house mobilization, there were a lot of Media attention program both at the radio and TV.” (KII/KT/SHE)*

#### Implementation

##### Data management

The HPV vaccination campaign employed a rigorous data management approach involving multiple personnel and comprehensive validation processes. Data collection was meticulous, with at least two team members recording client details immediately after vaccination using both paper and digital tools. The validation process was multi-leveled, starting at the ward level and progressing to the state level, with senior officers continuously checking and refining records to ensure data quality. This systematic approach allowed for real-time assessment, feedback gathering, and continuous improvement of the vaccination campaign's data management strategy.

*“You know, when they are vaccinating, they have tally sheets. So, the recorder, once the vaccinator is vaccinating one person, after vaccinating, the recorder will record. So then, you know, they have tally sheets. They also have a vaccine register, where the clients’ names appear, then they will transfer it to their cards, then to the tally sheet. Then from there, when they get to their facility at the ward level, the Ward Focal person will also cross-check everything that they have done before bringing it to the local government.” (IDI/EK/Ido LG/LHE)*

*“So, they have the tally sheets with them, they have also the summary sheets with them. So, after vaccinating a child they are expected to tally, the vaccinator is expected to tally and then at the end of the day they come together as a team and then they summarize their work into the summary form. That would be submitted to the ward focal person in which the ward focal person will also collate for all the teams under her/him and then the ward focal person will go and also transfer that to the ward level summary sheet and then that will also be submitted at the LGA for this LGA summary and after the LGA summary will also enter that into the call in template by the STFS and the M&E and that will be forwarded to the states for collation for the states.” (KII/EK/SIO)*

*“…one of the major validating processes for this is based on the vaccine utilization. So just like I said, Gardasil is a one dose vial. And the good thing is when you vaccinate one person, I will know you vaccinated one person. So we validated the vaccination report based on the data we have about the state and utilization of the vaccine. So it's tied to that. You know, you can't tell me you've vaccinated a hundred thousand and you've only utilized 60 of your vaccine. I mean, I would question that. Okay. So, I tie that down to the vaccine utilization and I know, of course, and then before the data even reaches the past two courses, I'm sure, I'm sure what you're vaccinating because they are being monitored every day. Remember during the MAC campaign, we have the daily evening review meetings happening where you give a report of what you're doing every day. So if you start bringing up issues that I do not understand, I'll tackle it from that angle.” (KII/National HPVVI Focal Person)*

##### Funding and financial management

The HPV vaccine introduction was financially supported through a collaborative effort between government and multilateral agencies. A comprehensive approach to fund management was implemented, with direct disbursements to activity implementers, detailed accountability measures, and transparent tracking of expenses. Funds were allocated to specific activities like healthcare worker allowances, social mobilization, and operational costs, with states providing supplementary funding to address budget gaps. The program benefited from both monetary and non-monetary support, including logistical resources, security arrangements, and contributions from private individuals and community leaders. Funding guidelines included developing work plans, resource mapping, and establishing clear protocols for fund allocation. Payments were made directly to beneficiaries and responsible officers, ensuring proper resource management and comprehensive program execution.

*“Like support that comes through the Health Educator for the ACSM activities, already there is always a voucher, when you are meeting with the stakeholders or the traditional leaders, already there is always a voucher that after the meeting they will basically sign. Attendance is there, they will take the attendance and sign, whatever is meant to be given to who it’s meant for is given and the person signs and at the end of the day the social mobilization officer retire everything to the organization that supported” (IDI/PL/Bakin Ladi LGA/LIO)*

*“We were giving retirement. If we say we buy minerals, we have to give receipts. If we say we buy biscuits, we have to give receipt. Definitely, you have to send a picture of the activity that you are conducting at the time. We had to bring back attendance, those people that attended the activity. That is the way we accounted for the funds.” (IDI/KT/Katsina LGA/Gabas1/WFP)*

*“The fund from GAVI is contained in the VIG, which is the Vaccine Introduction Grant, which is the grant given by GAVI for all the countries that are eligible, like Nigeria, that have introduced similar things. And those funds were domiciled with two of our partners, WHO and UNICEF, not with my agency, not with the NPHCDA. And the WHO and UNICEF are responsible in terms of disbursing these funds based on accepted guidelines by GAVI. So, the funds were not domiciled here at the agency. However, all the activities that were in our plans that were funded, the funds were disbursed by WHO and UNICEF.” (KII/National HPVVI Focal Person)*

*“So, what we did was that we developed a comprehensive work plan, and we did a resource mapping where we sat down in the TWG meeting after we have outlined all the activities that require funding. For instance, if UNICEF has sent money for social mobilization to be utilized in the activity lineup, we put the funding that is coming from UNICEF on that.” (KII/KW/Director PHCDA)*

##### Service delivery

To deliver vaccines to the target population, Schools served as key vaccination sites, with teachers actively involved as crowd controllers and supporters to ensure girls felt comfortable. This vaccination exercise also happened early in the morning to avoid disrupting school activities. Temporary fixed posts were set up in communities to ensure accessibility, especially for out-of-school female children. While mobile teams moved across settlements and markets to vaccinate eligible girls and reach remote areas. In addition, there were religious and community engagements, announcements were made in churches and mosques to raise awareness and vaccinate eligible girls during the campaign, quranic schools were specifically targeted to include female children in religious settings. Lastly, extra vaccine doses were carried to ensure no insufficiencies during field activities.

*“we announced in churches and mosques, also I took extra vaccine along with me to the field to avoid insufficiency. There are some settlements you will go and meet girls on playground that are eligible.” (IDI/KW/Ifelodun LGA/RIO)*

*“They used temporary fixed posts. They cannot stay in one place that you should be coming to them, so they had to use temporary fixed posts. When they get to a settlement, the mobilizers will call people, and when they see that they’re done with the people there, they move to another area. That is the temporary fixed post that was used” (IDI/KW/Ifelodun LGA/LIO)*

##### Health workforce

The vaccination teams were formed at the LGA level to ensure adequate vaccination teams, including vaccinators, recorders, and social mobilizers. Daily implementation plans were developed for each team to ensure structured deployment to schools, health facilities, and community sites. The fixed post teams were stationed at health facilities or specific locations for walk-in vaccinations, mobile teams moved from one settlement to another to vaccinate out-of-school girls and those in remote areas, while the school teams visited primary and secondary schools to vaccinate eligible female children, ensuring high coverage of the target age group. Effective coordination and supervision of vaccination teams were carried out daily, teams gathered at the health centres each morning for instructions and to pick vaccines that will be used, the quantity given was based on population density and ward-specific needs.

*“In the morning all the teams gathered at the health center and they are given instructions on how to operate in the field. Yes, they all had a schedule, after they gather at the health center and we have instructed them, they all go to their scheduled locations.” (KII/EB/Ebonyi/Echi-Aba/WFP)*

### Challenges encountered during HPV vaccine introduction

The HPV vaccination campaign in Nigeria encountered numerous planning and implementation challenges. Key issues included insufficient funding, competing priorities, and inadequate planning at the state level. These constraints led to delays in schedule, limited preparation time, and smaller-than-optimal teams, which complicated the overall rollout of the vaccination program. Service delivery was significantly impacted by multiple obstacles, including harassment of vaccination teams, widespread misinformation, and community resistance. Rumors about vaccine safety, particularly false claims about fertility damage, were propagated by various actors, including some healthcare practitioners. Religious leaders and school administrators often refused or discouraged vaccination, creating additional barriers to reaching the target population. Data management and financial challenges further complicated the campaign. Technical issues such as poor connectivity, lack of proper internet infrastructure, and insufficient technological resources hindered data reporting. Financial constraints included delayed fund disbursement, insufficient allowances for teams, and economic inflation that increased operational costs. These multifaceted challenges required innovative strategies, community engagement, and persistent efforts to overcome resistance and successfully implement the HPV vaccination program.

*“Yeah, some of the challenges encountered was that planning at the sub-national level was not adequately done on time and that led to the extension of the introduction date of the vaccine. If you recall, last year we wanted to introduce the vaccines in September but because of poor planning at the sub-national level, the introduction period was extended…” (KII/Director ACSM)*

*“… there were a lot of rumour here and there of negative effects of the vaccine. They interpreted it negatively that it is going to do harm and destroy the genital of the female, it is going to serve as contraceptive & … So, the team, mobilization team educated that this is a vaccine that is going to serve as a protection of cervical cancer…” (IDI/KT/Katsina LGA/LHE)*

*“Another major challenge for us was more misconceptions, misinformation that mostly pushed through social media during the implementation. We have groups that came up very strongly to kick against HPV vaccination. That kind of pushed us back a bit, because funny enough, these groups came up very strongly to an- extent that they were having messages in different languages just to discourage people from taking this vaccine. They go online, they pick some controversial issues that they don't have clarity on, and they frame it in such a way to make the vaccine look as if we're out to hurt the Nigerian population.” (KII/National HPVVI Focal Person)*

*“…high level hesitancy from the part of religious leaders. There was this particular church, that the religious leader said, all the church members must not take it that it is evil agenda, things like that.” (KII/EKST/CSO 2/Coordinator)*

*“The challenge I can say that some of the data tools were not enough. There are some data tools that were not adequate like summary sheets.” (KII/EB/SIO)*

*“There was a time that I give one team vaccines, they came back and told me the vaccine is finished. But when I checked and counted their tally sheet, it was short of vaccinated clients. But after going through the register, we found some clients they didn't mark on the tally sheet.” (IDI/KT/Kurfi/Birchi/WFP)*

*“… sometimes we did have challenges, especially regarding network, you know while you are sending the information you have to use network, so sometimes we had problem of network.” (KII/KT/SHE)*

*“The challenge is that…we don't have enough computers at that level, we don't have enough computers” (IDI/EK/Ido LG/MOH)*

*“…there are places where you can't reach without bike (motorcycle). No car can go there, no tricycle. Before you reach the place where you can get bike, you’d have to trek about 30 min, maybe, so because of this we had inconveniences for reporting data in time.” (IDI/KT/Katsina/Kudu3/WFP)*

*“Electricity was a major problem…electric power was not constant. So, we had to burn diesel and then there was no funding for that.” (KII/CRS/SIO)*

*“one of the major issues we had with HPV is that the budget was made some time ago. Unfortunately, during the time of implementation, that is when the country was facing this high inflation rates, where the subsidy for oil has been removed. So that kind of tripled everything in terms of the transport costs, the feeding and stuff like that. But this is, we're also working with a limited budget here.” (KII/National HPVVI Focal Person)*

### Achievements, innovative practices, and lessons learned

Achievements

1. High Coverage Rates: The vaccination campaign achieved unprecedented coverage, with reports indicating that over 2 million girls were vaccinated across two phases, which is a significant milestone.
2. Increased Awareness: There was a notable increase in awareness about cervical cancer among women, particularly young women, who previously had limited knowledge about the disease and its implications. Many expressed a willingness to seek screening after gaining this knowledge.
3. Community Acceptance: Efforts to educate the community about the HPV vaccine helped to dispel myths regarding its effects on fertility, leading to increased acceptance and participation in the vaccination program.

Innovative Practices

1. Social Mobilization: Early and proactive social mobilization activities were initiated, even in the absence of state support. This included advocacy visits and community engagement that began months before the vaccination campaign. Also, vaccine champions were selected in the communities, these were influencers who the community listened to and they helped in debunking rumours about the vaccine.
2. Local Language Communication: Translating health messages into local dialects proved effective in gaining community trust and acceptance of the vaccine.
3. Collaboration with CSOs: Engaging Civil Society Organizations (CSOs) in the vaccination process was a new approach that enhanced outreach and education efforts, ensuring that local communities were well-informed and involved.
4. School Essay Competition: There was an essay competition in some states, this helped the schoolgirls to participate and champion the HPV campaign.

Lessons Learned

1. Importance of Early Planning: The necessity of starting sensitization and mobilization efforts early was emphasized, as it helped to prepare the community and reduce hesitancy.
2. Effective Rumor Management: Utilizing existing networks for rumor management and crisis communication was crucial in addressing misinformation about the HPV vaccine, which helped maintain public confidence.
3. Involvement of Influential Community Members: Collaborating with teachers, youth leaders, and other influential figures in the community was identified as a key strategy for successful campaigns, as their involvement helped to amplify the message and encourage participation.

*“Yes, it was the fact that the people accepted the vaccine. We were able to deal with vaccine hesitancy, As they were convinced to take the vaccine and they did hence it was successful” (KII/EB/Ebonyi Echi-Aba/WFP)*

*“The people were aware of the burden of cervical cancer, and this encouraged them to allow their wards take the vaccine. So, we also achieved awareness of cervical cancer.” (KII/PL/CSO2/Executive Director)*

*“One of the lessons learnt is early social mobilization activity. We actually kick start the social mobilization activity early enough despite the fact that we don't have support from the state. We started our advocacy visits very early as far as back as January… end of January, February, March, April we had a long number of months that we started social mobilization activity at the state even without support. So, is one of the lessons learnt because it actually helped us greatly so going forward for any other campaigns and vaccine introduction, it is a lesson that we have.” (KII/EK/SIO)*

*“Well, the success we have achieved is we vaccinated our target and even beyond the target because, I think by day 4 or day 5, if I could remember, our vaccine had finished, and we had to request for additional ones from the state.” (IDI/KT/Kurfi/Birchi/WFP)*

*“Now, the biggest of all the achievements was the overall percentage we got from the exercise I'm sure you know that Plateau State ran one of the top best” (KII/PL/DDCI)*

*“So, you see, most of the eligible clients, they were all vaccinated. In fact, we have to even look for additional vaccines from other places.” (IDI/KT/Katsina LGA/LCCO)*

*“Yeah, so for us, we felt the achievement is that the transparency of the fund disbursement is proof to even our partners that we can be trusted. We can be trusted to implement what we said we're going to do because the funds were not domiciled here, they were domiciled with our partners. So, it was a very clear, transparent process, clear to the whole world. I mean, there were documentations to show the funds, what these funds were used for by the partner that was of course holding these funds.” (KII/National HPVVI Focal Person)*

## Summary

In summary, a table was used to synthesise key findings from the HPV Phase II vaccination campaign report (Table 2). It presents each thematic area, the insights generated, lessons learned, and innovative practices.

**Table 2:** Thematic Analysis of Strategies, Insights, Lessons, and Innovations from the HPV Phase II Vaccination Campaign in Nine Nigerian States

Thematic area	Key insights	Innovative practice	Lessons learnt
1. Planning and coordination	TWG ensured multi-stakeholder collaboration Microplanning with GIS improved targeting	Expanded TWG included religious and community leaders Subcommittees for logistics, M&E	Early planning prevents delays Security collaboration critical in high-risk areas
2. Stakeholder engagement	Local influencers (teachers, traditional leaders) boosted trust Misinformation was a major barrier	Vaccine champions at ward level Jingles in native languages; school essay competitions	Early sensitization reduces hesitancy Rumors must be debunked swiftly via trusted networks
3. Financial management	Transparent funds via WHO/UNICEF Inflation impacted budgets	Activity-based budgeting with receipts Resource mapping to align funds	Direct disbursement ensures accountability State-level co-funding fills gaps
4. Supply chain and logistics	Buffer stocks prevented shortages Poor roads delayed deliveries	"Hit-and-run" vaccinations in security compromised areas Mobile teams with motorcycles	Flexible logistics needed for hard-to-reach areas
5. Service delivery	Schools as key sites (teachers as facilitators) Temporary posts reached out-of-school girls	Quranic school inclusion Early-morning sessions to avoid disruption	Community-based posts improve access
6. Health workforce	Mixed teams (vaccinators, recorders) enhanced efficiency Understaffing due to budgets	Daily team briefings at health centers	Training cascades ensure skill retention
7. Data management	Real-time validation Poor internet hindered reporting	Hybrid paper-digital systems Multi-level data checks	Offline tools essential for remote areas Invest in LGA-level tech (tablets)
8. Supervision	Daily review meetings tracked progress On-the-spot corrections	State validation of LGA microplans	Continuous monitoring improves data quality
Achievements	2 million + girls vaccinated (exceeded targets in some states) Increased cervical cancer awareness	CSO collaboration expanded outreach	Community ownership drives success

## Discussion

For the first time in Nigeria’s history, the nation successfully implemented the HPV vaccine introduction for girls aged 9–14 and its eventual inclusion in the country’s Routine Immunization (RI) schedule. With millions of girls vaccinated and more taking the vaccine post-campaign at the health facilities, the campaign's success is owed to conscious efforts, adequate preparation, and support from different parties. To ensure localized advocacy, it was crucial to include traditional leaders, religious leaders, town criers, and community-based mobilizers. Similar results have been documented in vaccination acceptance where leveraging community trust and religious leaders were crucial factors in vaccination acceptability [26, 27]. The necessity of customized communication to successfully reach a variety of populations was further underscored by the translation of campaign messaging into local languages. This strategy is consistent with data indicating that culturally relevant messaging greatly increases vaccination uptake in diverse groups [28].

The findings from our study showed that the establishment of a Technical Working Group (TWG) made it easier to include a variety of stakeholders and promoted cooperation and a sense of shared responsibility, to debunk rumors and gain support, advocacy trips to leaders in the fields of education and religion were crucial. According to a study on the importance of partnerships in vaccination programs, these initiatives highlight the value of a multi-stakeholder approach in fostering an environment that supports vaccine campaigns [29]. Both in-school and out-of-school girls had access to the vaccine, this was achieved because of the campaign's utilization of schools as immunization sites, temporary posts in communities, and mobility teams for hard-to-reach areas. These strategies, which highlight the importance of flexibility in reaching target populations, are in line with best practices in vaccination distribution [30]. In addition, the creation of microplans and thorough training cascades were essential to maximizing labor productivity and resource allocation. The study also showed that the use of technology may improve operational planning and monitoring, as demonstrated by the campaign's dependence on GIS mapping and real-time data validation. In a study in Pakistan, the authors have demonstrated how well data-driven approaches may be integrated to enhance immunization results [31]. Through innovative financial management strategies, the campaign garnered substantial attention despite budgetary limitations. The utilization of both monetary and non-monetary resources and community leaders' logistical assistance highlights how crucial it is to have a variety of funding sources to maintain health programs.

The HPV vaccination campaign in Nigeria, brought to light the complex connections between operational, community, and structural issues that might impede public health campaigns. The campaign had many challenges despite its notable coverage, which emphasizes the necessity of careful preparation, clear communication, and flexible approaches in vaccination rollout initiatives. Significant obstacles that led to delays and poor planning were a lack of finances and conflicting priorities at the state level. These difficulties are consistent with more general problems observed in low-resource vaccination programs, where it has been demonstrated that budgetary limitations lessen the efficacy of implementation strategies [32]. Furthermore, operational difficulties were made worse by inflation and postponed payments of allowances to the vaccination teams, underscoring the significance of prompt and sufficient resource allocation to enable thorough campaign implementation. From the study, another challenge to vaccination uptake was the spread of false information, especially myths regarding the safety of vaccines and their effects on fertility. Similar results have been noted in other low- and middle-income nations, where cultural beliefs, false information, and mistrust of health authorities are major factors contributing to vaccine hesitancy [33–36]. To overcome these challenges, customized communication approaches that involve reliable local influencers and offer factual, culturally aware information to debunk myths and foster public confidence are necessary [37]. Another challenge that was encountered was the disruption of service delivery in some communities by harassment of vaccination teams and insufficient team sizes as a result of financial limitations. These problems show that to guarantee the security and effectiveness of vaccination teams, better workforce planning and improved security measures are required. Furthermore, the fact that certain religious and community leaders refused to allow vaccination in their areas emphasizes how crucial it is to engage stakeholders early before implementation to overcome resistance and bring community leaders on board [38].

Despite the challenges encountered during the vaccination campaign, significant milestones were achieved, highlighting the value of creative approaches and community involvement in enhancing public health outcomes. One key achievement was the vaccination of approximately three million girls who received the HPV vaccine during the campaign across the nine intervention states, marking a significant milestone in the fight against cervical cancer. This achievement demonstrates how well data-driven strategies and broad community involvement work to achieve high vaccination rates. Additionally, the campaign raised awareness of cervical cancer, especially among young women, many of whom said they were now more inclined to get screened. Innovative strategies that tackled communication and logistical issues further contributed to the campaign's success. In preparation for the campaign, proactive social mobilization was crucial in promoting community acceptability. In line with suggestions from earlier vaccination efforts in Africa, the use of vaccine champions and influencers to dispel myths and promote the HPV vaccine proved very successful in combating misinformation and generating demand [37]. By translating health messages into local languages, various groups' confidence and understanding were greatly increased, which further decreased vaccine hesitancy. Furthermore, a strategy that increased uptake and strengthened community collaborations was the campaign's incorporation of Civil Society Organizations (CSOs), this is in agreement with some studies that underscored the importance of CSOs involvement in vaccination campaigns [39, 40], additionally, schoolgirls were empowered to become champions for the HPV when a school essay competition was introduced this fostered enthusiasm for the program.

Finally, key lessons were learned during the HPV vaccine introduction across the nine states, the initiative yielded important insights that emphasized the value of community sensitization and early planning months before the campaign to guarantee proper planning, reduce hesitancy, and promote higher participation. The study also found that resolving misinformation and preserving public trust in the vaccine required efficient rumor control and crisis communication, which were made possible by pre-existing community networks. A key factor in the campaign's success was the participation of powerful community members, such as educators, young leaders, and religious, and traditional leaders, who promoted acceptance and amplified messages. These lessons reaffirm the importance of community-driven approaches in overcoming barriers to immunization efforts [26].

The study draws its strengths from the application of a variety of strategies, such as community engagement, social mobilization, and communication in the local language, which offered insightful information about practical methods for raising vaccination rates in diverse and resource-constrained settings. The study also shows the impact and scalability of well-coordinated vaccination campaigns, with almost three million girls that were vaccinated. The involvement of community influencers ensured tailored interventions that addressed cultural sensitivities and community-specific issues. Lastly, new strategies that encouraged community ownership and advocacy, like school essay contests and partnerships with Civil Society Organizations (CSOs), added a unique dimension to the campaign.

### Limitations

The study’s scope was limited to nine states, excluding insights from other implementing states involved in the second phase of the vaccine introduction. Additionally, the study focused solely on campaign outcomes, without assessing the long-term effects of the campaign on cervical cancer prevention or sustained vaccine uptake. Furthermore, funding limitations, workforce shortages, and operational challenges, highlighted in the study, may have affected the quality and comprehensiveness of the campaign’s implementation and data collection. The study also did not examine potential inequities in access to vaccination, such as differences between in-school and out-of-school girls or between rural and urban populations. Future research could explore these disparities to better understand their impact on vaccination coverage. Notably, a recent paper has identified disparities and inequities associated with HPV mass vaccination in Nigeria [41] and proposed the adoption of a gender-neutral and single-dose policy, which has significantly improved coverage across various levels of the Cameroonian health system since 2023 [42].

### Policy recommendation

Based on findings from this study, the following recommendations should be considered. First, to avoid implementation delays, guarantee sufficient staff capacity, and meet operational needs, especially in environments with limited resources, sustainable and timely funding should be given priority. To build confidence and lessen resistance, future vaccination efforts should place a high priority on early engagement with local influencers, religious leaders, and traditional leaders. Mechanisms to evaluate the long-term effects of HPV vaccination campaigns, such as variations in the incidence of cervical cancer and sustained vaccine uptake, should be incorporated into future research. This would offer more thorough proof of the program's accomplishments as well as its shortcomings. Furthermore, to quickly combat disinformation, it is essential to develop strong rumor management systems that make use of current community networks, such as social media platforms. Myths can also be debunked by educating medical professionals on the safety and effectiveness of the vaccine on an ongoing basis. Lastly, adequate allowances and field team safety are critical to campaign success, and comprehensive training programs should be put in place to address issues like harassment and operational inefficiencies.

## Conclusion

The successful introduction of the HPV vaccine in Nigeria marks a significant milestone in public health, particularly for the prevention of cervical cancer among girls aged 9–14. This initiative is not just a health intervention; it represents a collaborative effort involving various stakeholders, including government agencies, community leaders, and health workers, all dedicated to safeguarding the future of Nigeria's young women. The campaign's success can be attributed to meticulous planning and stakeholder engagement. By identifying key players and fostering partnerships across national and local levels, the program effectively raised awareness and dispelled misinformation about the vaccine. Training healthcare workers ensured they were well-equipped to administer the vaccine and address community concerns, thereby enhancing trust in the initiative. Despite facing challenges such as rumors and logistical issues, the commitment of all involved parties led to the achievement of vaccination goals. The proactive measures taken to counter misinformation and the emphasis on community involvement were crucial in mobilizing support for the vaccination campaign. Nigeria's HPV vaccination initiative serves as a model for future health campaigns as it highlights the importance of collaboration, community engagement, and responsive strategies in overcoming barriers to public health interventions.

## Supplementary Information

Below is the link to the electronic supplementary material.

Supplementary Material 1.

## Data Availability

The datasets used and/or analyzed during the current study are available from the corresponding author upon reasonable request. However, the interview guide is available as supplementary material.

## Abbreviations

ACSM: Advocacy, Communication, and Social Mobilization
CCAE: Cervical Cancer Action for Elimination
CSOs: Civil Society Organizations
EPI: Expanded Program on Immunization
eTWG: Expanded Technical Working Group
FCT: Federal Capital Territory
HCWs: Health Care Workers
HPV: Human papillomavirus
LMIC: Low- and Middle-Income Country
LGA: Local government area
MAC: Multi-age cohort
MP: Micro plan
NCCP: National Cancer Control Plans
NPHCDA: National Primary Health Care Development Agency
NHREC: National Health Research and Ethics Committee
SSA: Sub-Saharan Africa
TWG: Technical Working Group
