# Supplementary material for: Stakeholders’ perspectives on lessons learnt from HPV mass vaccination in Nigeria
Source: BMC Public Health. 2025 Sep 24;25:3062. doi: 10.1186/s12889-025-24418-0 (PMC12462204; doi:10.1186/s12889-025-24418-0)
Supplement: Supplementary file 1 — Supplementary Material 1. [file 12889_2025_24418_MOESM1_ESM.docx]

**Key Informant Interview Guide for National & State-level Officers**

KII No.: **________**

**Informed Consent (Oral)**

Good day Sir/Ma, my name is _____________________ and I work for Sydani Group. My organization is currently undertaking a study titled “**HPV Vaccine Introduction: Lessons Learned and Future Directions from the Vaccination Intervention in Nine (9) Nigerian States”**. This study seeks to document and analyze the implementation strategies, achievements, challenges, and lessons learned from the HPV VI Phase II project, and to proffer recommendations that could be used to improve prospective vaccine introductions and inform policymaking. I would appreciate it if you could spare some of your time to answer some questions. I assure you that all information shared with me shall be kept in utmost confidentiality. Although the interview is voluntary and you have permission to exit at any time, I would appreciate it if you could complete the interview. Please note that this interview session will be recorded to document what is being discussed adequately.

Do I have your permission to go ahead with the interview? Yes/No

*(End the interview if no, and continue if yes)*

**SECTION A: Socio-demographics**

1. **Please, introduce yourself.**

Focus: *Prompt where the participant skips any of the following*

- Gender
- Level of education
- Age range (<30, 30-39, 40-49, 50-59, 60-69, 70-79, >79)
- Designation
- Number of years serving in that designation
- Role played during the HPV vaccination exercise

**SECTION B: Activities conducted during the HPV Vaccine Introduction**

1. **Planning and Coordination**

- Kindly describe to us your understanding of the HPV Technical Working Group (TWG)

***Prompt***: *Who were the members of the TWG?* *Their responsibilities (****Probe****: if all the members have the same responsibilities or not)*

- Kindly tell us in detail, what role the TWG meetings played in the state’s readiness for the vaccine introduction

***Prompt****: Frequency of the meetings held. Activities and/or discussion involved (****Probe****: list of the activities and their explanation)*

- What led to the development of the expanded Technical Working Group (eTWG)? And What can you tell us about it?

***Prompt:*** *Who were the members, and what were their responsibilities? (****Probe****: do they all have the same responsibilities or not*

- Please share with us, your knowledge of the Microplan developed for the vaccine introduction.

***Prompt:*** *Importance of development. Processes (from beginning to end) and players involved in the development. Evaluation and validation of the MP (****Probe****: The validation process, the persons responsible)*

- What are the achievements of the planning and coordination process (***Prompt:*** *TWG and eTWG meetings, microplanning*)
- Tell us about the challenges and how they were managed or addressed

1. **Stakeholder Engagement/ACSM**

- Please tell us about the identification process and engagement of stakeholders (EPI and non-EPI, including the CSOs) for the vaccine introduction.

***Prompt****: How were they (i) identified (including CSOs), and (ii) engaged*

- What were the contributions of the identified stakeholders to the vaccine introduction?

***Probe:*** *How would you describe their impact on the vaccine introduction?*

- What can you tell us about the ACSM strategies adopted for promoting vaccine introduction across the state?

*(****Probe****: (i) Specific activities, (ii) promotion channels across all levels, people and locations), iii) Responsible people, (iv) Impact of the activities, (v)* effectiveness of the promotional *activities (****Prompt****:* *Approach used to monitor and evaluate the promotional activities*)

- What were the challenges encountered in engaging stakeholders and the promotional activities for vaccine introduction *(****Probe:*** *focus on challenges in managing CSOs and CBOs)*
- What roles did the stakeholders play in creating awareness and uptake of the vaccine?
- Kindly describe the successes recorded in stakeholders’ engagement & promotional activities from CSOs and/or CBOs

1. **Financial Management/Funding**

- What approaches were utilized to generate funds and resources for the vaccine introduction and how were they utilized?

***Prompt****: (i) Sources (i.e organizations), (ii) fund types, (iii) Purpose fund served (i.e activities, teams/individuals’ payment etc.),*

***Probe****:* What other forms of support were provided? And what were the sources?

- What can you tell us about the accountability and transparency system put in place for funds management.

***Prompt:*** *(i) Responsible people for disbursement, (ii) processes for adequate and effective fund allocation*

- What type of compensations were provided to the vaccination teams? *(****Probe****: The frequency of payment, the people paid)*
- What achievements were recorded from your adopted approach in funds management/disbursement
- What were the challenges encountered, and how did you manage to address them

1. **Supply Chain & Logistics**

- What can you tell us about the availability & functionality of the cold chain equipment throughout the campaign?
- How would you describe the sufficiency of the vaccination equipment? (vaccine carriers, cold boxes etc)
- Did you receive enough HPV vaccine, and how were you able to manage the distribution of the vaccines?
- How were the vaccines and other equipment distributed to the relevant sites? (**Probe**: *The distribution plan, officers involved in the development of distribution plan, distribution process*)
- What were the infrastructures put in place to ensure the security of the vaccination equipment? (**Probe**: *The equipment used to secure and maintain the vaccines at appropriate temperature, how data tools and other equipment were safeguarded*)
- What achievements were recorded in the measures you adopted for optimal distribution
- What were the challenges encountered, and how did you manage to address them

1. **Service Delivery and Health Workforce**

- Kindly tell us about the recruitment process involved in the vaccination exercise

***Prompt:*** *(i) recruitment strategy (ii) categories of recruited personnel (HCW/Non-HCW), (iii), team composition*

***Probe:*** *Were* the recruited personnel enough for the vaccine introduction?

- What form(s) of training(s) was conducted for the healthcare workers and how was the trainings conducted? (**Probe**: *When the training(s) took place, the levels of training, personnel trained across levels*)
- Please tell us in detail the strategies adopted to deliver vaccines to the target population

**Prompt**: (i) *The different types of vaccination sessions/strategies, (ii) method of deployments (of each strategy), (iv) What you consider novel or innovative in the strategies*

- How did the vaccination team manage the wastes generated during vaccination? (**Probe**: *The waste collation, collection, and incineration processes, persons responsible*)
- What achievements were recorded from the adopted approach
- What were the challenges encountered, and how did you manage to address them

1. **Supervision**

- What supervision strategy was deployed during the vaccination exercise?

***Prompt****: (i) How many levels of supervision, (ii) Responsible people at each level, (iii) supervisory duties at each level, and (iv) Frequency of supervision?*

- What achievements were recorded from this approach
- What were the challenges encountered, and how did you manage to address them

1. **Data Management**

- Kindly describe the data collection process at the vaccination sites

**Prompt**: (i) *Persons in charge of data recording, (ii) the types of data collected, (iii) Data collection tools (iv)*

- Kindly explain the reporting flow for the collected data. (**Probe**: *The reporting process from the vaccination teams to the appropriate stations, and officers involved in the reporting process*)
- What were the validation processes for the vaccination data? (**Probe**: *explanation of the processes, and officers responsible*)
- What achievements were recorded from this approach
- What were the challenges encountered, and how did you manage to address them

**SECTION C: Lessons learned and innovative practices**

1. What were other innovative strategies adopted or implemented during the HPV vaccine introduction? (Probe: The things done differently that positively impacted the project)
2. What were the lessons learned during the HPV vaccine introduction? (Probe: Major failure, what could have been done better)

**Conclusion**

1. What recommendations do you have for future HPV vaccine introduction and other related vaccine?
2. What recommendations do you have for routinizing the new vaccine?

*Thank you for your time*

**In-Depth Interview Guide for**

**Local Government and Ward Level Officers in the State**

IDI No.: ________

**Informed Consent (Oral)**

Good day Sir/Ma, my name is _____________________ and I work for Sydani Group. My organization is currently undertaking a study titled “ **”**. The study seeks to document and analyze the implementation strategies, achievements, challenges and lessons learned from the HPVVI Phase II project, and to proffer recommendations that could be used to improve prospective vaccine introductions and inform policymaking. I would appreciate it if you could spare some of your time to answer some questions. I assure you that all information shared with me shall be kept in utmost confidentiality. Although the interview is voluntary and you have permission to exit at any time, I would appreciate it if you could complete the interview. Please note that this interview session will be recorded to document what is being discussed adequately.

Do I have your permission to go ahead with the interview? Yes/No

*(End the interview if no, and continue if yes)*

**SECTION A: Socio-demographics**

1. **Please, introduce yourself.**

Focus: *Prompt where the participant skips any of the following*

- Gender
- Level of education
- Age range (<30, 30-39, 40-49, 50-59, 60-69, 70-79, >79)
- Designation
- Number of years serving in that designation
- Role played during the HPV vaccination exercise

**SECTION B: Activities conducted during the HPV Vaccine Introduction**

1. **Planning and Coordination**

- Kindly describe to us your understanding of the HPV Technical Working Group (TWG)

***Prompt***: *Who were the members of the TWG? Their responsibilities (****Probe****: if all the members have the same responsibilities or not)*

- Kindly tell us in detail, what role the TWG meetings played in the state’s readiness for the vaccine introduction

***Prompt****: Frequency of the meetings held. Activities and/or discussion involved (****Probe****: list of the activities and its explanation)*

- What led to the development of the expanded Technical Working Group (eTWG)? And What can you tell us about it?

***Prompt:*** *Who were the members, What were their responsibilities? (****Probe****: do they all have the same responsibilities or not*

- Please share with us, your knowledge of the Microplan developed for the vaccine introduction?

***Prompt:*** *Importance of development. Processes (from beginning to end) and players involved in the development. Evaluation and validation of the MP (****Probe****: The validation process, the persons responsible)*

- What are the achievements of the planning and coordination process (***Prompt:*** *TWG and eTWG meetings, microplanning*)
- Tell us about the challenges and they were managed or addressed

1. **Stakeholder Engagement/ACSM**

- Please tell us about the identification process and engagement of stakeholders (EPI and Non EPI, including the CSOs) for the vaccine introduction?

***Prompt****: How were they (i) identified (including CSOs), and (ii) engaged*

- What were the contributions of the identified stakeholders to the vaccine introduction?

***Probe:*** *How would you describe their impact on the vaccine introduction?*

- What can you tell us about the ACSM strategies adopted for promoting vaccine introduction across the state?

*(****Probe****: (i) Specific activities, (ii) promotion channels across all levels, people and locations), iii) Responsible people, (iv) Impact of the activities, (v)* effectiveness of the promotional *activities (****Prompt****:* *Approach used to monitor and evaluate the promotional activities*)

- What were the challenges encountered in engaging stakeholders and the promotional activities for vaccine introduction *(****Probe:*** *focus on challenges in managing CSOs and CBOs)*
- What roles did the stakeholders play in creating awareness and uptake of the vaccine?
- Kindly describe the successes recorded stakeholders’ engagement & promotional activities from CSOs and/or CBOs

1. **Financial Management/Funding**

- What approaches were utilized to generate funds and resources for the vaccine introduction and how were they utilized?

***Prompt****: (i) Sources (i.e organizations), (ii) fund types, (iii) Purpose fund served (i.e activities, teams/individuals’ payment etc.),*

***Probe****:* What other forms of support were provided? And what were the sources?

- What can you tell us about the accountability and transparency system put in place for the funds management.

***Prompt:*** *(i) Responsible people for disbursement, (ii) processes for adequate and effective fund allocation*

- What type of compensations were provided to the vaccination teams? *(****Probe****: The frequency of payment, the people paid)*
- What achievements were recorded from your adopted approach in funds management/disbursement
- What were the challenges encountered, and how did you manage to address them

1. **Supply Chain & Logistics**

- What can you tell us about the availability & functionality of the cold chain equipment throughout the campaign?
- How would you describe the sufficiency of the vaccination equipment? (vaccine carriers, cold boxes etc)
- Did you receive enough HPV vaccine, and how were you able to manage the distribution of the vaccines?
- How were the vaccines and other equipment distributed to the relevant sites? (**Probe**: *The distribution plan, officers involved in the development of distribution plan, distribution process*)
- What were the infrastructures put in place to ensure the security of the vaccination equipment? (**Probe**: *The equipment used to secure and maintain the vaccines at appropriate temperature, how data tools and other equipment were safeguarded*)
- What achievements were recorded in the measures you adopted for optimal distribution
- What were the challenges encountered, and how did you manage to address them

1. **Service Delivery and Health Workforce**

- Kindly tell us about the recruitment process involved in the vaccination exercise

***Prompt:*** *(i) recruitment strategy (ii) categories of recruited personnel (HCW/Non HCW), (iii), team composition*

***Probe:*** *Were* the recruited personnel enough for the vaccine introduction?

- What form(s) of training(s) was conducted for the healthcare workers and how were the trainings conducted? (**Probe**: *When the training(s) took place, the levels of training, personnel trained across levels*)
- Please tell us in detail the strategies adopted to deliver vaccines to the target population

**Prompt**: (i) *The different types of vaccination sessions/strategies, (ii) method of deployments (of each strategy), (iv) What you consider novel or innovative in the strategies*

- How did the vaccination team manage the wastes generated during vaccination? (**Probe**: *The waste collation, collection and incineration processes, persons responsible*)
- What achievements were recorded from the adopted approach
- What were the challenges encountered, and how did you manage to address them

1. **Supervision**

- What supervision strategy was deployed during the vaccination exercise?

***Prompt****: (i) how many levels of supervision, (ii) Responsible people at each level, (iii) supervisory duties at each level, and (iv) Frequency of supervision?*

- What achievements were recorded from this approach
- What were the challenges encountered, and how did you manage to address them

1. **Data Management**

- Kindly describe the data collection process at the vaccination sites

**Prompt**: (i) *Persons in-charge of data recording, (ii) the types of data collected, (iii) Data collection tools (iv)*

- Kindly explain the reporting flow for the collected data. (**Probe**: *The reporting process from the vaccination teams to the appropriate stations, officers involved in the reporting process*)
- What were the validation processes for the vaccination data? (**Probe**: *explanation of the processes, officers responsible*)
- What achievements were recorded from this approach
- What were the challenges encountered, and how did you manage to address them

**SECTION C: Successes recorded during the vaccine introduction**

1. What were other innovative strategies adopted or implemented during the HPV vaccine introduction? (Probe: The things done differently that positively impacted the project)
2. What were the lessons learned during the HPV vaccine introduction? (Probe: Major failure, what could have been done better)

**Conclusion**

1. What recommendations do you have for future HPV vaccine introduction and other related vaccine?
2. What recommendations do you have for how to routinize the new vaccine?

*Thank you for your time*

**In-Depth Interview Guide for CSOs**

IDI No.: **________**

**Informed Consent (Oral)**

Good day Sir/Ma, my name is _____________________ and I work for Sydani Group. My organization is currently undertaking a study titled “**HPV Vaccine Introduction: Lessons Learned and Future Directions from the Vaccination Intervention in Nine (9) Nigerian States”**. This study seeks to document and analyze the implementation strategies, achievements, challenges, and lessons learned from the HPV VI Phase II project, and to proffer recommendations that could be used to improve prospective vaccine introductions and inform policymaking. I would appreciate it if you could spare some of your time to answer some questions. I assure you that all information shared with me shall be kept in utmost confidentiality. Although the interview is voluntary and you have permission to exit at any time, I would appreciate it if you could complete the interview. Please note that this interview session will be recorded to document what is being discussed adequately.

Do I have your permission to go ahead with the interview? Yes/No

*(End the interview if no, and continue if yes)*

**SECTION A: Socio-demographics**

1. **Please, introduce yourself.**

Focus: *Prompt where the participant skips any of the following*

- Gender
- Level of education
- Age range (<30, 30-39, 40-49, 50-59, 60-69, 70-79, >79)
- Name of Organization
- Designation /Title
- Role played during the HPV vaccination exercise
- Level of operation (***Prompt:*** *if they were engaged for state- or LGA-level activities*)

**SECTION B: Activities conducted during the HPV Vaccine Introduction**

1. **Planning and Coordination**

- Were you a member of the eTWG? Yes/No
- What were your responsibilities as a member of the eTWG? ***Probe****: the roles they played, the planning and coordination activities they were responsible for*
- How would you describe the impact of planning and coordination on the vaccine introduction?
- What are the achievements of the planning and coordination process (***Prompt:*** *eTWG meetings, microplanning*)
- Tell us about the challenges encountered and how they were managed or addressed

1. **Stakeholder Engagement/ACSM**

- Please describe your role in awareness creation and stakeholder engagement during the HPV vaccination.

***Probe****: How were they engaged, the activities they were responsible for, and the approach*

- What were your contributions during the vaccine introduction?

***Probe:*** *How would you describe the impact of your activities on the vaccine introduction?*

- What can you tell us about the ACSM strategies adopted for promoting vaccine introduction across the state?

*(****Probe****: (i) Specific activities, (ii) promotion channels across all levels, people and locations), iii) Responsible people, (iv) Impact of the activities, (v)* effectiveness of the promotional *activities (****Prompt****:* *Approach used to monitor and evaluate the promotional activities*)

- Kindly describe the achievements recorded due to awareness creation and promotional activities
- What were the challenges encountered, and how did you manage to address them?

**Conclusion**

1. What recommendations do you have for future HPV vaccine introduction and other related vaccine?
2. What recommendations do you have for routinizing the new vaccine?

*Thank you for your time*
